# Pregenual or subgenual anterior cingulate cortex as potential effective region for brain stimulation of depression

**DOI:** 10.1002/brb3.1591

**Published:** 2020-03-08

**Authors:** Ying Jing, Na Zhao, Xin‐Ping Deng, Zi‐Jian Feng, Guo‐Feng Huang, Meng Meng, Yu‐Feng Zang, Jue Wang

**Affiliations:** ^1^ Institutes of Psychological Sciences Hangzhou Normal University Hangzhou China; ^2^ Zhejiang Key Laboratory for Research in Assessment of Cognitive Impairments Hangzhou China; ^3^ Center for Cognition and Brain Disorders and the Affiliated Hospital Hangzhou Normal University Hangzhou China; ^4^ School of Information and Electronics Technology Jiamusi University Jiamusi China

**Keywords:** depression, functional connectivity, pregenual anterior cingulate cortex, repetitive transcranial magnetic stimulation, subgenual anterior cingulate cortex

## Abstract

**Background:**

The dorsolateral prefrontal cortex (DLPFC) is the standard stimulation target for the repetitive transcranial magnetic stimulation (rTMS) treatment of major depression disorder (MDD). A retrospective study by Fox and colleagues found that a more negative resting‐state functional magnetic resonance imaging (RS‐fMRI) functional connectivity (FC) between left DLPFC and the subgenual anterior cingulate cortex (sgACC) in a large group of healthy participants is associated with a better curative effects of rTMS in MDD, suggesting that the sgACC may be an effective region. However, a recent meta‐analysis on RS‐fMRI studies found that the pregenual ACC (pgACC), rather than the sgACC, of MDD patients showed increased local activity.

**Methods:**

We used the stimulation coordinates in the left DLPFC analyzed by Fox et al. to perform RS‐fMRI FC between the stimulation targets obtained from previous rTMS MDD studies and the potential effective regions (sgACC and pgACC, respectively) on the RS‐fMRI data from 88 heathy participants.

**Results:**

(a) Both the pgACC and the sgACC were negatively connected to the left DLPFC; (b) both FCs of sgACC‐DLPFC and pgACC‐DLPFC were more negative in responders than in nonresponders; and (c) the associations between DLPFC‐sgACC functional connectivity and clinical efficacy were clustered around the midline sgACC.

**Conclusions:**

Both the pgACC and the sgACC may be potential effective regions for rTMS on the left DLPFC for treatment of MDD. However, individualized ACC‐DLPFC FC‐based rTMS on depression should be performed in the future to test the pgACC or the sgACC as effective regions.

## Graphical abstract

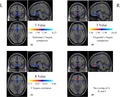

## 1 INTRODUCTION

Repetitive transcranial magnetic stimulation (rTMS) has been approved by the FDA (food and drug administration) for depression treatment. In past years, meta‐analyses showed that application of high‐frequency rTMS over the left dorsolateral prefrontal cortex (DLPFC) had antidepressant effect (Berlim, Frederique, & Daskalakis, 2013; Burt, Lisanby, & Sackeim, 2002; Kedzior & Reitz, 2014; Lesenskyj, Samples, Farmer, & Maxwell, 2018; Slotema, Blom, Hoek, & Sommer, 2010). The left DLPFC was selected as a stimulation target in light of a previous neuroimaging study, which showed decreased glucose metabolism in the left DLPFC in patients with MDD (Baxter et al., 1989). The general method, namely “5‐cm rule,” of locating DLPFC was established in 1995 (George et al., 1995). It consists of locating the hotspot in the left primary motor cortex first, then moving 5 cm anteriorly in the parasagittal plane, presumably targeting the left DLPFC. Although it is a convenient way to locate the DLPFC stimulation target, the “5‐cm rule” does not account for individual variability of brain size and morphology, potentially resulting in studies finding no significant stimulation effects (Herbsman et al., 2009; Herwig, Padberg, Unger, Spitzer, & Schönfeldt‐Lecuona, 2001). Moreover, the locations of the hand motor hotspot are varied largely in population (Ahdab, Ayache, Brugieres, Farhat, & Lefaucheur, 2016), and it makes the target location defined by “5‐cm rule” more heterogeneous. Investigators started to notice about the importance of precisely localizing of stimulation target (Battelli, Grossman, & Plow, 2017; Eldaief, Halko, Buckner, & Pascual‐Leone, 2011; Wang et al., 2014).

Fox, Buckner, White, Greicius, and Pascual‐Leone (2012) proposed that the rTMS stimulation on the left DLPFC was related to a deep brain region named subgenual anterior cingulate cortex (sgACC). To test their hypothesis, they performed functional connectivity (FC) of the sgACC on a dataset of resting‐state functional magnetic resonance imaging (RS‐fMRI) from 98 healthy young adults. The authors found that the left DLPFC targets which were reported in previous studies (Fitzgerald et al., 2009; Herbsman et al., 2009; Paillere Martinot et al., 2010) with stronger negative FC of sgACC‐DLPFC were associated with better efficacy (Fox et al., 2012). Based on these evidences, they concluded that the stimulation on the DLPFC may take antidepression effects through the sgACC‐DLPFC network. Such remote effect on deep brain region via stimulating on superficial cortex has also been reported in rTMS studies (Arfeller et al., 2013; Lazzaro et al., 2011; Nahas et al., 2001; Solomon‐Harris, Rafique, & Steeves, 2016). For simplicity, we hereafter called the superficial cortex target as “stimulation target,” and correspondingly called the deep brain region as “effective region.”

Fox et al. (2012) listed a few evidences for taking the sgACC as an effective region for rTMS treatment of MDD. One evidence was that the regional cerebral blood flow (rCBF) in the sgACC decreased after TMS treatment (Kito, Fujita, & Koga, 2008; Kito, Hasegawa, & Koga, 2011). Another evidence showed that the sgACC was a successful target for deep brain stimulation (DBS) for depression treatment (Drevets, Savitz, & Trimble, 2008). However, some studies showed that the pregenual ACC (pgACC) is also a pivotal brain region for MDD (Ken‐Ichi & Graybiel, 2012; Mannie et al., 2008). The pgACC region consistently showed elevated rCBF during the episode of major depressive disorder (Drevets, 2000), and significant increases of glucose and lactate are associated with depression severity (Ernst et al., 2016; Sacher et al., 2012). Apart from that, there are ample evidences elucidated that the increased pretreatment pgACC activity (the theta activity of electroencephalogram signal, the rCBF, the activation of fMRI signal during simple task and so forth) predicts better antidepressant response after kinds of treatment (TMS, medicine, sleep deprivation and so forth) (Pizzagalli, 2010). Another magnetic resonance spectroscopy (MRS) study found decreased glutamate and glutamine ratio in the pgACC in MDD patients (Horn et al., 2010). More recently, a coordinate‐based meta‐analysis study on RS‐fMRI found that the pgACC of patients with MDD had increased amplitude of low frequency fluctuation (ALFF) (Zhou et al., 2017). Therefore, based on the above multi‐modal imaging studies, the pgACC might also be a potential effective region for treatment of MDD. To this end, the current study hypothesized that the FC values in the pgACC also have certain association with clinical improvement. We test this hypothesis by investigating the anticorrelation between the pgACC and the DLPFC in a large group of healthy participants and related the pgACC‐DLPFC anticorrelation to the reported clinical efficacy of rTMS, similarly as did by Fox et al. (2012). The results would help us to understand the brain mechanism of rTMS treatment on MDD.

## 2 MATERIALS AND METHODS

### 2.1 Data composition

There were two datasets in the current study. Dataset 1 was RS‐fMRI from 88 young healthy adults (Elaborated on below). Dataset 2 was some data from published studies, including: (a) the coordinates of the sgACC (Fox et al., 2012) and the pgACC (Zhou et al., 2017) (Table 1); (b) the left DLPFC coordinates of rTMS targets (better efficacy target and less efficacy target) (Table 1); (c) the clinical improvement and the corresponding left DLPFC target coordinates of 27 patients (Paillere Martinot et al., 2010) (Table 2); and (d) the nine DLPFC sites from previous studies (Table 3). All the coordinates were in the Montreal Neurological Institute (MNI) space.

**Table 1:** The coordinates of ACC subregions and four TMS stimulation targets in the DLPFC

	*X*	*Y*	*Z*
ACC coordinates (MNI)			
Subgenual ACC (Fox et al., 2012)	6	16	−10
Pregenual ACC (Zhou et al., 2017)	0	42	6
DLPFC coordinates			
Responders' target (Herbsman et al., 2009)	−46	23	49
Nonresponders' target (Herbsman et al., 2009)	−41	17	55
More effective target (Fitzgerald et al., 2009)	−46	45	38
The converted more effective target	−39	40	31
Less effective target (Fitzgerald et al., 2009)	−41	16	54

Note

To be noticed, the coordinates of more effective target which recorded by Fitzgerald (Fitzgerald et al., 2009) were located out of the brain cortical area, so we projected this coordinate to the nearest cortex.

Abbreviations: ACC, anterior cingulate cortex; DLPFC, dorsal lateral prefrontal cortex; MNI, Montreal neurological institute.

**Table 2:** The coordinates of subject‐specific targets and the corresponding clinical efficacy from a TMS study (Paillere Martinot et al., 2010) as well as the mean FC values with ACC subregions

Subject	MNIx	MNIy	MNIz	MADRS % Improvement	Mean FC values^a^			
					5‐mm pgACC	5‐mm sgACC	10‐mm pgACC	10‐mm sgACC
01	−46	26	26	46.34	−0.20	−0.12	−0.23	−0.14
02	−18	56	28	78.38	0.16	0.02	0.22	0.05
03	−36	36	42	69.57	−0.07	−0.08	−0.06	−0.09
04	−4	66	8	6.98	0.33	0.13	0.41	0.17
05	−38	32	−6	70	0.05	0.01	0.07	0.01
06	−14	58	34	14.81	0.16	0.01	0.21	0.05
07	−48	36	16	93.94	−0.14	−0.10	−0.16	−0.12
08	−36	40	36	91.49	−0.09	−0.07	−0.11	−0.08
09	−32	28	54	68	−0.05	−0.06	−0.03	−0.06
10	−40	13	56	28	−0.10	−0.08	−0.09	−0.08
11	−37	17	57	62.07	−0.07	−0.07	−0.05	−0.07
12	−43	22	49	50	−0.10	−0.09	−0.09	−0.09
13	−45	23	50	64.29	−0.08	−0.08	−0.06	−0.08
14	−38	23	48	52.63	−0.09	−0.09	−0.08	−0.09
15	−42	18	53	82.35	−0.10	−0.08	−0.08	−0.08
16	−51	4	42	73.17	−0.20	−0.07	−0.22	−0.08
17	−44	22	43	78.38	−0.14	−0.10	−0.14	−0.11
18	−39	14	58	72.22	−0.09	−0.08	−0.08	−0.07
19	−45	2	50	95	−0.17	−0.10	−0.18	−0.11
20	−45	30	41	64	−0.14	−0.11	−0.15	−0.13
21	−49	33	29	40	−0.20	−0.12	−0.23	−0.14
22	−45	33	41	−12.5	−0.13	−0.11	−0.13	−0.12
23	−52	25	35	42.42	−0.17	−0.11	−0.19	−0.12
24	−35	14	63	25.81	−0.06	−0.06	−0.06	−0.06
25	−37	14	58	6.98	−0.08	−0.08	−0.08	−0.07
26	−47	28	39	27.27	−0.16	−0.12	−0.18	−0.13
27	−49	20	46	29.73	−0.14	−0.10	−0.14	−0.11

Abbreviations: ACC, anterior cingulate cortex; FC, functional connectivity; MADRS, Montgomery‐Åsberg Depression Rating Scale; MNI, Montreal neurological institute; pgACC, pregenual ACC; sgACC, subgenual ACC; TMS, transcranial magnetic stimulation.

^a^ Mean FC values represent the average ACC‐DLPFC FC values across 88 healthy participants at a certain DLPFC target.

**Table 3:** The DLPFC sites which reported in previous literatures and the corresponding estimated clinical efficacy, as well as the mean FC values toward with four sub‐ACCs

DLPFC Definition	MNIx	MNIy	MNIz	Estimated HDRS% Improvement^a^	Mean FC values^b^			
					5‐mm pgACC	5‐mm sgACC	10‐mm pgACC	10‐mm sgACC
Average 5‐cm Coordinate (Fox et al., 2012)	−41	16	54	0.254	−0.10	−0.10	−0.10	−0.09
Responders' target (Herbsman et al., 2009)	−46	23	49	0.448	−0.13	−0.12	−0.13	−0.11
Nonresponders' target (Herbsman et al., 2009)	−41	17	55	0.266	−0.10	−0.10	−0.09	−0.09
EEG (F3) Site (Herwig et al., 2003)	−37	26	49	0.286	−0.11	−0.11	−0.10	−0.10
BA46 (Rajkowska & Goldman‐Rakic, 1995)	−44	40	29	0.608	−0.11	−0.12	−0.12	−0.15
BA9 (Rajkowska & Goldman‐Rakic, 1995)	−36	39	43	0.42	−0.04	−0.11	−0.03	−0.12
TMS Target (Cho & Strafella, 2009)	−40	31	34	0.412	−0.14	−0.14	−0.14	−0.16
TMS Target (Rusjan et al., 2010)	−50	30	36	0.62	−0.20	−0.15	−0.21	−0.16
Converted Fitzgerald Target^c^	−39	40	31	0.498	−0.05	−0.11	−0.06	−0.14

Abbreviations: ACC, anterior cingulate cortex; BA, Brodmann area; DLPFC, dorsal lateral prefrontal cortex; EEG, electroencephalogram; FC, functional connectivity; HDRS, Hamilton Depression Rating Scale; MNI, Montreal Neurological Institute; pgACC, pregenual ACC; sgACC, subgenual ACC; TMS, transcranial magnetic stimulation.

^a^ The estimated HDRS% improvement was derived from an empirical equation reported by Herbsman et al. (2009).

^b^ Mean FC values represent the average ACC‐DLPFC FC values across 88 healthy participants at a certain DLPFC target.

^c^ The converted Fitzgerald target was derived from the coordinate (*x* = −46, *y* = 45, and *z* = 38) which reported by Fitzgerald et al. (2009). Because the original coordinate locates out of brain, we converted it to the cortex and created the new coordinate (*x* = −39, *y* = 40, and *z* = 31).

### 2.2 Data collection and analyses

#### 2.2.1 Participants

Eighty‐eight healthy young adults (43 female, age = 23.2 ± 2.9) with no history of neurological or psychiatric disorders were recruited. The present research was approved by the Ethics Committee of the Center for Cognition and Brain Disorders (CCBD) at Hangzhou Normal University. Written informed consent was signed by each subject before the experiment.

#### 2.2.2 Data acquisition

All subjects underwent 8‐min RS‐fMRI scans in a 3T scanner (MR‐750, GE Medical Systems) with the following parameters: slice number = 43, matrix size = 64 × 64, FOV = 220 × 220 mm, TR/TE = 2,000/30 ms, FA = 90 deg, slice thickness/gap = 3.2/0 mm, and voxel size = 3.4 × 3.4 × 3.2 mm^3^. During scanning, participants were instructed to keep their eyes closed and not to fall asleep. A high‐resolution T1 weighted image was acquired by spoiled gradient recalled 3‐D MRI sequence (slice number = 176, matrix size = 256 × 256, FOV = 256 × 256 mm, TR/TE = 8.1/3.1 ms, FA = 8 deg, slice thickness/gap = 1/0 mm, and voxel size = 1 × 1 × 1 mm^3^).

#### 2.2.3 Data preprocessing

RS‐fMRI data were preprocessed using the Statistical Parametric Mapping (SPM12, http://www.fil.ion.ucl.ac.uk/spm) software and DPABI toolbox (http://rfmri.org/dpabi) (Yan, Wang, Zuo, & Zang, 2016) on the MATLAB platform. All the preprocessing procedures were consistent with those in Fox et al.s' (2012) study, included the following procedures: (a) removal of the first 10 volumes; (b) slice timing correction; (c) head motion correction; (d) co‐registration of T1 image to EPI image; (e) segmentation; (f) normalization by using T1 image; (g) smoothing (Gaussian kernel of full‐width half maximum, FWHM = 6 mm); (h) band‐pass filtering (0.009–0.08 Hz); and (i) nuisance regression (head motion effects with six movement parameters by rigid body translation and rotation, linear trends, white matter, cerebrospinal fluid, and global mean time course).

#### 2.2.4 Whole‐brain voxel‐wise FC of the sgACC and the pgACC

**Figure 1:**
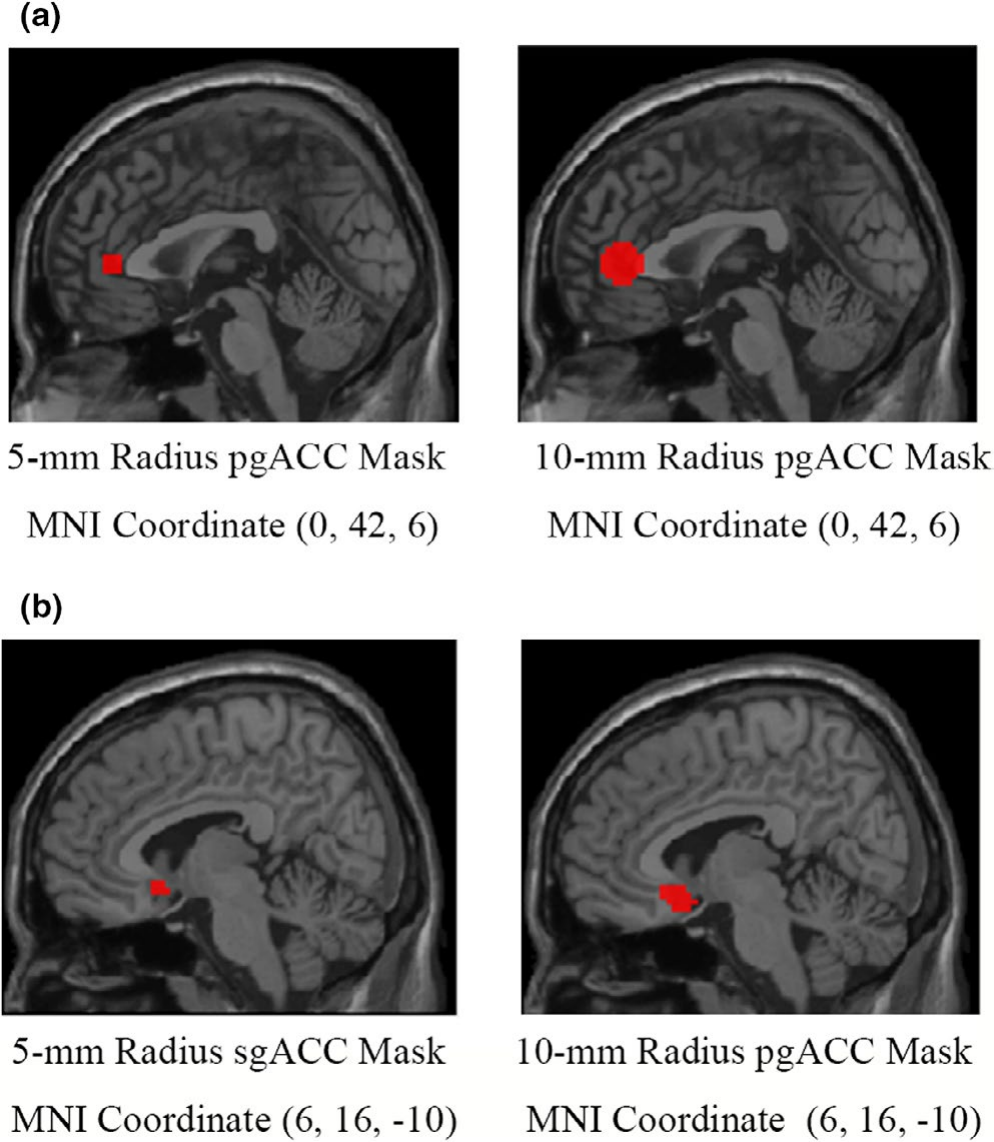
The seed regions of interest (ROI) of the pgACC and the sgACC. The 5‐mm and 10‐mm radius spheres for both the sgACC and the pgACC were generated by centering on the coordinates determined by previous studies (Fox et al., 2012; Zhou et al., 2017). ACC, anterior cingulate cortex; pgACC, pregenual ACC; sgACC, subgenual ACC

The seed ROIs were placed at the pgACC and the sgACC separately. The sgACC coordinate (*x* = 6, *y* = 16, and *z* = −10) was selected from the study by Fox et al. (2012). The pgACC coordinate (*x* = 0, *y* = 42, and *z* = 6) was obtained from an RS‐fMRI meta‐analysis in which increased ALFF in the pgACC was reported in depressive patients (Zhou et al., 2017) (Table 1). Two kinds of radius (5 and 10 mm) for the ROIs were used in the current study. We used the 10‐mm radius ROI in order to keep consistent with Fox et al. (2012). Considering that the sgACC and the pgACC are not far from each other anatomically, a 10‐mm radius ROI could probably increase the similarity of their time courses. We therefore also used a 5‐mm radius ROI. A gray matter probability threshold of 0.25 was used on the Harverd/Oxford cortical template (http://www.cma.mgh.harvard.edu/) (Figure 1) to remove the white matter and cerebrospinal fluid in the seed ROIs.

The average time course was extracted from each ROI. To generate FC map, Pearson's correlation coefficients were computed for each of the four ACC seed ROIs (the sgACC of 5‐ and 10‐mm, and the pgACC of 5‐ and 10‐mm, respectively) in a voxel‐wise way through the whole brain. Fisher's *r*‐to‐*z* transformation was applied for each correlation coefficient to fit the normal distribution.

After the calculation of FC, one sample *t* tests were performed on *z*‐FC maps to reveal the FC pattern of ACC ROIs in the whole brain. The FDR (false discovery rate) correction (*q* < 0.001, cluster size > 100 voxels) was used to produce robust statistical maps.

#### 2.2.5 ACC‐DLPFC FC comparisons between stimulation targets

Fox et al. (2012) selected two previous studies of rTMS on MDD, each study reporting two DLPFC stimulation targets, that is, responder's target versus nonresponder's target (Herbsman et al., 2009) and more effective target versus less effective target (Fitzgerald et al., 2009), respectively, from previous MDD TMS treatment studies (Table 1). Fox et al. calculated the FCs between the sgACC and four DLPFC targets as mentioned above in a group of 98 healthy participants. They found that the stimulation targets with better efficacy showed stronger negative FC than the targets with worse efficacy (Fox et al., 2012).

We then repeated Fox's comparisons on 88 healthy participants of our data. To be noticed, Fitzgerald's coordinates of the more effective target (*x* = −46, *y* = 45, and *z* = 38) were located out of the brain (Fitzgerald et al., 2009). Fox et al. used a large radius of 20 mm to include cortex. Recently, Ji, Yu, Liao, and Wang (2017) proposed a method to project the scalp TMS target to cortical surface and successfully applied by Wang et al. (2019), so we used the same method and projected the original coordinate to the cortex as follows. First, we calculated the nearest cortex to the original coordinates, then went 6 mm deeper in cortical region, and got the converted more effective target (*x* = −39, *y* = 40, and *z* = 31) (Ji et al., 2017; J. Wang et al., 2019). Different from Fox et al., the following process used the new converted effective target.

In the RS‐fMRI data of 88 healthy participants, we performed ROI‐wise FC between ACC and DLPFC. A 20‐mm radius spherical ROI was centered at the four DLPFC targets, respectively (responders and nonresponders in Herbsman's study, as well as less effective target and converted more effective target in Fitzgerald's study), as did by Fox et al. (2012). A gray matter probability threshold of 0.7 was used on the Harverd/Oxford cortical template to ensure all the voxels in the DLPFC ROIs are within the gray matter. The ACC ROIs were centered at the sgACC and the pgACC, respectively, each with a 5‐mm radius and a 10‐mm radius (see Section 2.2.4 for details). For each participant, the mean time course of each ROI was extracted; then, Pearson's correlation coefficient was computed.

To compare the FC strength, 3‐way repeated‐measure analysis of variance (ANOVA) with stimulation target (better efficacy vs. worse efficacy), the type of ACC (pgACC vs. sgACC), and type of radius (5 vs. 10 mm) as main factors was performed for Herbsman's targets and Fitzgerald's targets separately (please see the Supporting Information for details).

#### 2.2.6 Correlation between ACC‐DLPFC FC of 27 stimulation targets and clinical efficacy of 27 patients

Paillere Martinot et al. (2010) performed a rTMS study and reported the coordinates of 27 individual stimulation targets in the left DLPFC as well as individual clinical efficacy (i.e., reduction of scores in Montgomery‐Åsberg Depression Rating Scale or MADRS) of 27 MDD patients (Table 2). In order to find out the potential relationship of TMS target and the clinical efficacy, Fox et al. calculated the correlation between the sgACC‐DLPFC FC strength (correlation coefficients) on the 27 DLPFC coordinates and the corresponding clinical efficacy. The results showed a strong negative correlation between the FC values and the clinical efficacy.

We replicated the above process as did by Fox et al. in our data of 88 healthy participants, but we included both the sgACC and the pgACC ROIs with two kinds of radius (5 and 10 mm). As did by Fox et al., we centered the 10‐mm DLPFC ROIs on 27 stimulation targets in Table 2. Using the Harverd/Oxford cortical template with an intensity of 0.7, the voxels lying outside of gray matter were eliminated (Fox et al., 2012). After that, the mean time course was extracted from each of the 27 10‐mm DLPFC ROIs and the four ACCs (sgACC and pgACC, both with 5‐ and 10‐mm radius). Then, we computed ROI‐wise FC between DLPFC targets and ACCs in our 88 healthy participants. All the FC values were averaged across the 88 participants, so every DLPFC target had one mean FC value representing the correlation between this target and each of the four ACC ROIs. Pearson's correlation coefficients between the ACC‐DLPFC FC strength and clinical efficacy were then calculated (*n* = 27).

#### 2.2.7 Correlation between ACC‐DLPFC FC of nine DLPFC sites and corresponding predicted clinical efficacy

In Fox's study, the authors selected nine DLPFC sites from seven studies (Table 3), and calculated the correlation between sgACC‐DLPFC FC (averaged across 98 healthy participants) and corresponding clinical efficacy as predicted by a previous reported equation: Hamilton Depression Rating Scale (HAM‐D) drop = −0.84 + (*X* × −0.022) + (*Y* × 0.012) (Herbsman et al., 2009). The correlation showed that more presumed effective DLPFC sites were more negatively correlated with the sgACC, that is, more anticorrelation of sgACC‐DLPFC was associated with better efficacy (Paillere Martinot et al., 2010).

We did the same correlation analyses between ACC‐DLPFC FC and predicted clinical efficacy on our 88 healthy participants' fMRI data. The nine DLPFC ROIs were the same as those of Fox's study except for one DLPFC site (*x* = −46, *y* = 45, and *z* = 38), which reside outside the brain (Fitzgerald et al., 2009). We converted it to the cortex as elaborated in Section 2.2.5 (Ji et al., 2017; Wang et al., 2019). In addition, for the ACC, we utilized four ACC ROIs (sgACC and pgACC, both with 5‐mm and with 10‐mm radius).

## 3 RESULTS

### 3.1 The voxel‐wise FC between ACCs and whole brain

**Figure 2:**
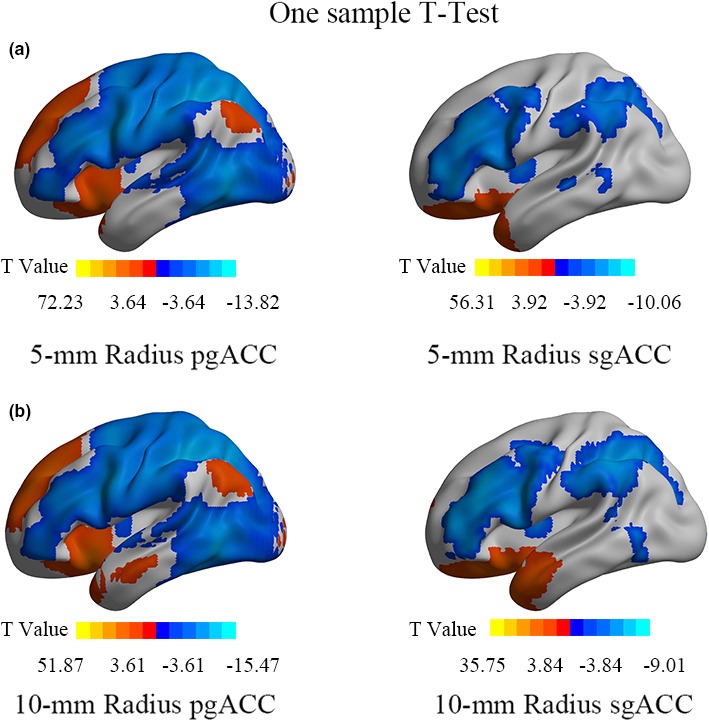
The FC patterns of the sgACC and pgACC with different size of seeds (a, b) (FDR correction, *q* < 0.001, cluster size > 100 voxels, two‐tailed). The warm color indicates positive FC of ACCs, and the cold color indicates negative FC. ACC, anterior cingulate cortex; FC, functional connectivity; pgACC, pregenual ACC; sgACC, subgenual ACC

Both the pregenual and subgenual ACCs were negatively connected with the DLPFC (threshold *q* < 0.001, cluster size > 100 voxels) (Figure 2).

### 3.2 ACC‐DLPFC FC comparisons

**Figure 3:**
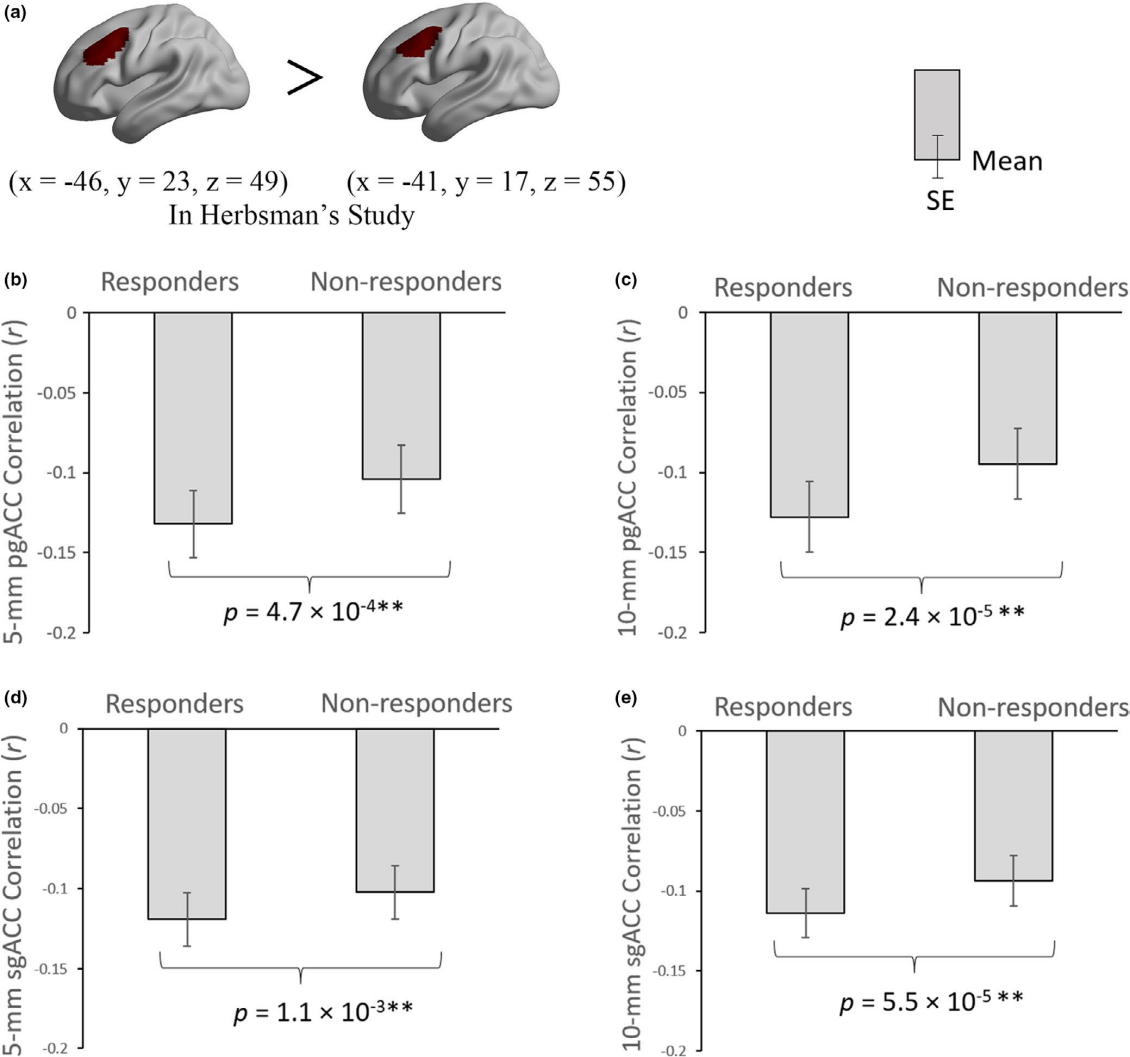
The differences of ACC‐DLPFC FCs between the responders and nonresponders. The DLPFC stimulation targets were from Herbsman et al. (2009). The colored regions in the brain indicate the ROIs of DLPFC (a). The definition of DLPFC was centered in the mean coordinate of responders and nonresponders with a 20‐mm radius, respectively. ACC, anterior cingulate cortex; DLPFC, dorsal lateral prefrontal cortex; FC, functional connectivity; pgACC, pregenual ACC; *SE*, standard error; sgACC, subgenual ACC. *p < .05; **p < .01

For the stimulation targets in Herbsman' study (Herbsman et al., 2009), ANOVA results revealed a significant main effect of target factor (*F*_1,87_ = 22.496, *p* = 8.0 × 10^–6^). After pairwise comparisons, we found both the sgACC and the pgACC showed significantly higher negative ACC‐DLPFC FC for the responders' DLPFC target than the nonresponders' DLFPC target (Table S2 and Figure 3). Please see the Supporting Information for the details of interaction effect and following simple effect analyses (Figure S2).

**Figure 4:**
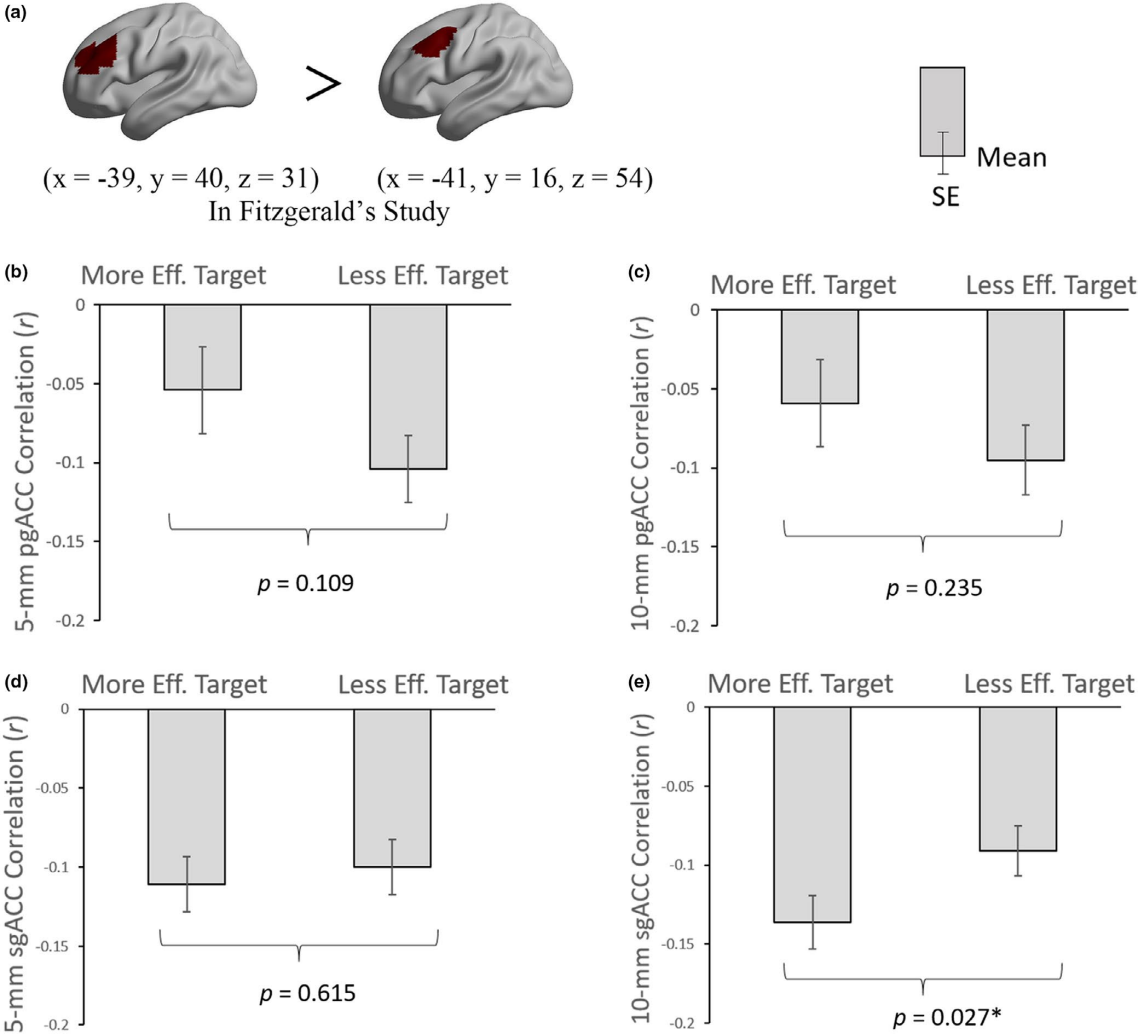
The differences of ACC‐DLPFC FCs between the more effective target and less effective target. The DLPFC stimulation targets were from Fitzgerald et al. (2009) and Fox et al. (2012). The colored regions in the brain indicate the ROIs of DLPFC (a). The definition of DLPFC was centered in the coordinate of more effective target and less effective target with a 20‐mm radius, respectively. ACC, anterior cingulate cortex; DLPFC, dorsal lateral prefrontal cortex; eff., effective; FC, functional connectivity; pgACC, pregenual ACC; *SE*, standard error; sgACC, subgenual ACC. *p < .05

Referring to Fitzgerald's pair of targets, the 3‐way repeated‐measure ANOVA revealed no significant main effect for any of the three factors, that is, the ACC‐DLPFC FC values in each pair of more effective versus less effective target, sgACC versus pgACC, and 5‐mm versus 10‐mm radius of ACC were basically similar (Table S2). The follow‐up pairwise comparison results showed that only the 10‐mm radius sgACC‐DLPFC FC had significant difference between more effective target and less effective target (*F*_1,87_ = 5.032, *p* = .027) (Figure 4). The results of interaction effect and following simple effect analyses can be seen in Supporting Information (Figure S3, S4).

### 3.3 Correlation between ACC‐DLFPC FC of 27 stimulation targets and corresponding clinical efficacy of 27 patients

**Figure 5:**
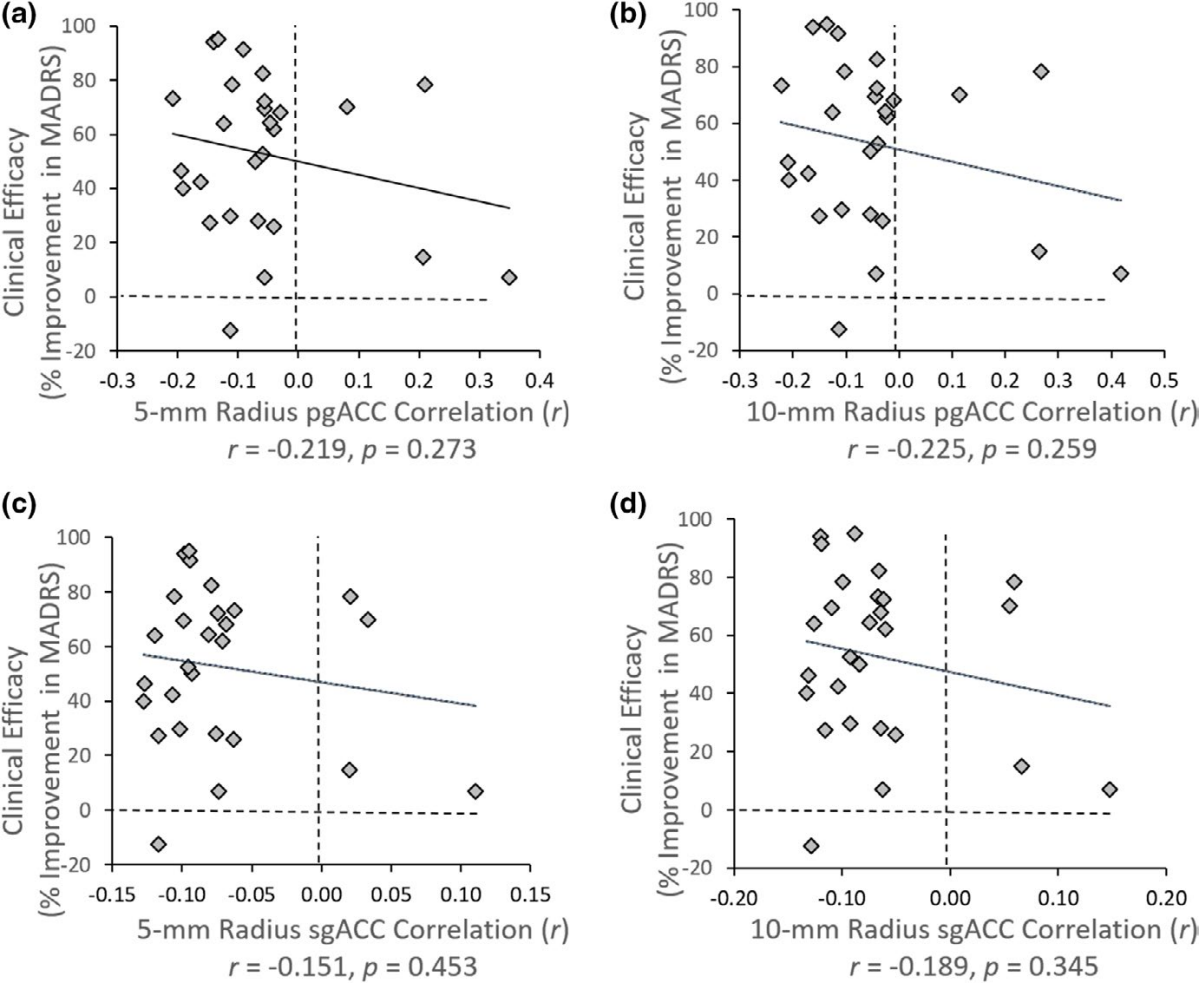
Correlation between 27 ACC‐DLPFC targets FCs and clinical efficacy of 27 patients. Every dot represents the mean FC value of ACC‐DLPFC across 88 healthy participants at a certain DLPFC target. The definition of DLPFC was centered in the previously reported TMS target with a 10‐mm radius. ACC, anterior cingulate cortex; DLPFC, dorsal lateral prefrontal cortex; FC, functional connectivity; MADRS, Montgomery Asberg Depression Rating Scale; pgACC, pregenual ACC; sgACC, subgenual ACC; TMS, transcranial magnetic stimulation

No significant correlation was found between ACC‐DLPFC FC of the 27 stimulation targets and corresponding clinical efficacy of the 27 patients on our RS‐fMRI dataset (mean FC of 88 healthy participants) (Figure 5) (*r*_5‐mm pgACC_ = −0.219, *p* = .273; *r*_10‐mm pgACC_ = −0.225, *p* = .259; *r*_5‐mm sgACC_ = −0.151, *p* = .453; *r*_10mmsg_ = −0.189, *p* = .345).

### 3.4 Correlation between ACC‐DLPFC FC of nine DLPFC coordinates and corresponding estimated clinical efficacy

**Figure 6:**
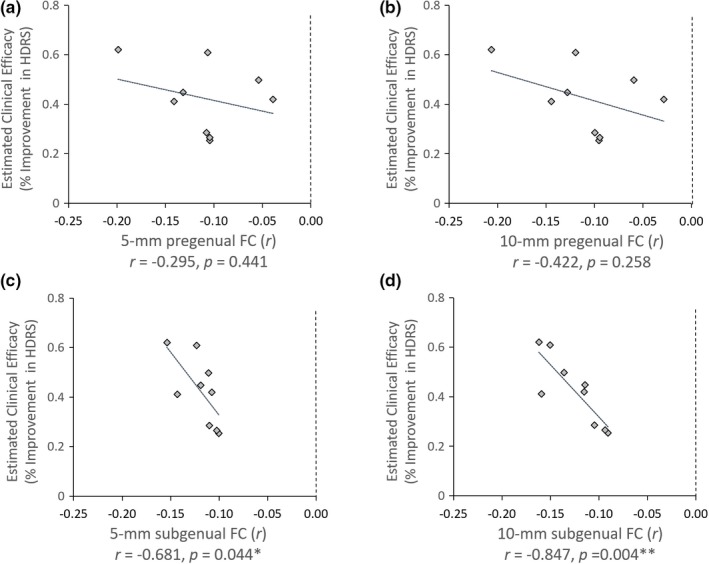
Correlation between estimated clinical efficacy of nine different DLPFC coordinates and functional connectivity of the sgACC/pgACC. The nine DLPFC targets were selected from literatures (see Table 3), and the estimated clinical efficacy was derived from an empirical equation reported by Herbsman et al. (2009). ACC, anterior cingulate cortex; DLPFC, dorsal lateral prefrontal cortex; FC, functional connectivity; HDRS, Hamilton Depression Rating Scale; pgACC, pregenual ACC; sgACC, subgenual ACC. *p < .05; **p < .01

There were significant anticorrelations between sgACC‐DLPFC FC (both in 5‐mm and in 10‐mm radius) and the corresponding estimated clinical efficacy of the nine DLPFC coordinates (Figure 6c,d) (*r*_5‐mm sgACC_ = −0.681, *p* = .044; *r*_10‐mm sgACC_ = −0.847, *p* = .004), whereas we did not find significant correlation in the pgACC (Figure 6a,b) (*r*_5‐mm pgACC_ = −0.295, *p* = .441; *r*_10‐mm pgACC_ = −0. 422, *p* = .258).

### 3.5 New analyses for sgACC‐DLFPC FC

The laterality of sgACC‐DLPFC FC seems to be a paradox: the sgACC coordinates (*x* = 6, *y* = 16, and *z* = −10) are in the right sgACC; however, the rTMS targets are in the left DLPFC. Furthermore, the radius of the sgACC is also a concern because, for Fitzgerald's pair of targets, the 10‐mm radius FC showed significant differences between more and less rTMS efficacy, but the 5‐mm did not (as shown in the Section 3.2). We supposed that the sgACC should be in the left side and that a 10‐mm radius of the seed ROI may cover heterogeneous functional areas. We thus performed new analyses as follows.

**Figure 7:**
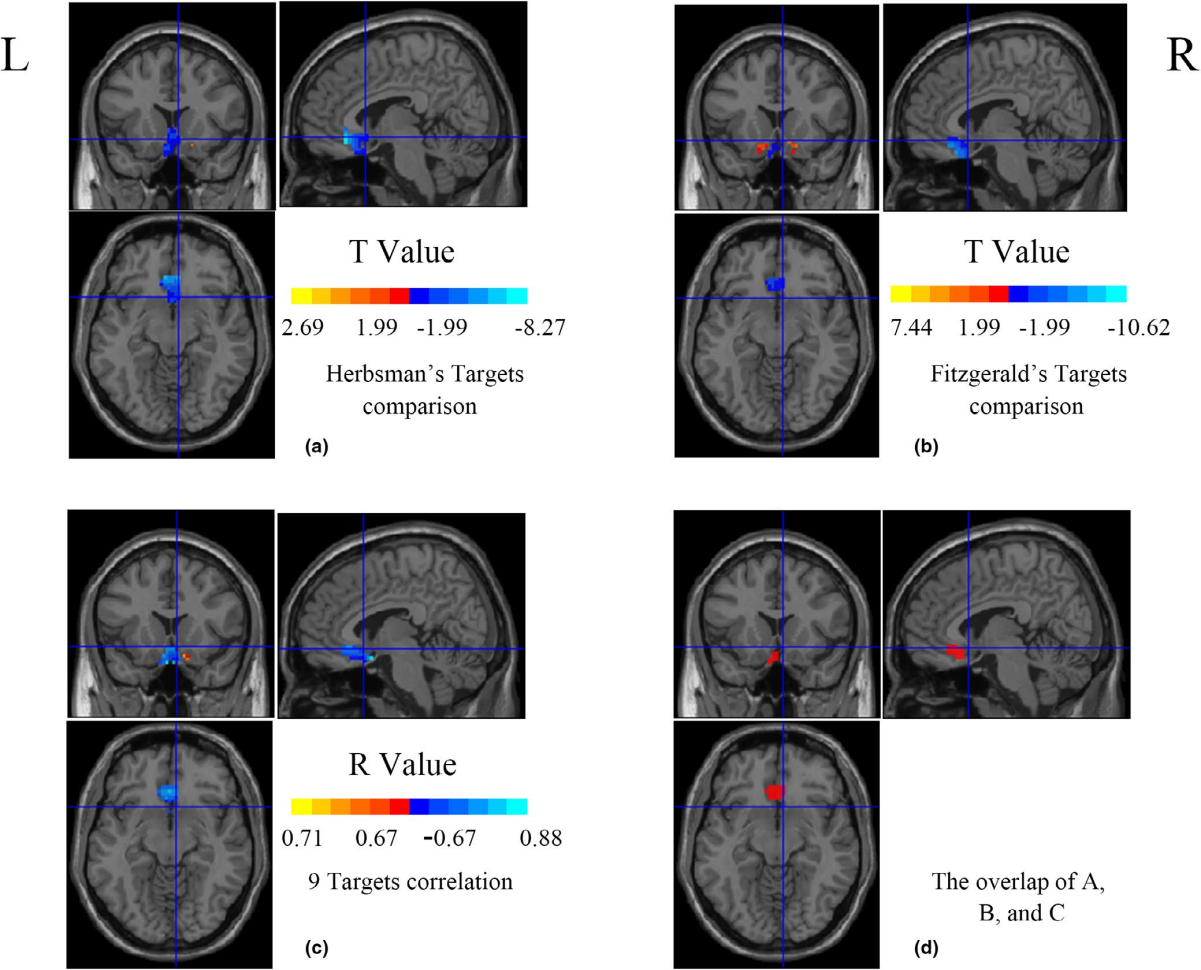
The voxel‐wise FC analyses between DLPFC and sgACC in a 20‐mm radius medial sgACC ROI (*x* = 0, *y* = 16, and *z* = −10). The crosshair located in the original sgACC coordinate (*x* = 6, *y* = 16, and* z* = −10) from Fox et al. (2012). The colored regions in a and b represent the differences between two DLPFC TMS targets (better efficacy target vs. less efficacy target). The cold color indicates that better clinical efficacy target showed more negative FC (a and b). The cold color region in c represents negative correlation between DLPFC‐sgACC FC and estimated clinical efficacy scores in nine DLPFC coordinates. All the statistical maps were thresholded at uncorrected *p* < .05. The red color in d represents overlapping brain region of a, b, and c. DLPFC, dorsal lateral prefrontal cortex; FC, functional connectivity; L, left; R, right; ROI, region of interest; sgACC, subgenual anterior cingulate cortex; TMS, transcranial magnetic stimulation

(a) We moved the coordinates (*x* = 6, *y* = 16, and *z* = −10) to the midline (*x* = 0, *y* = 16, and −*z* = 10) and defined a big spherical ROI with radius of 20 mm. Then the 20‐mm medial sgACC ROI was masked by 0.25 Harverd/Oxford cortical template to eliminate the voxels lying outside the gray matter. (b) We re‐performed the analyses of Sections 2.2.5 and 2.2.7 for each voxel in the medial sgACC ROI. We did not repeat Section 2.2.6 because neither the 10‐mm radius ROI nor the 5‐mm radius ROI showed significant correlation. (c) We set a *p* value of <.05 (uncorrected) for each of the three maps (*t* maps of Herbsman' targets FC comparison, *t* maps of Fitzgerald's targets FC comparison, and *r* map of ACC‐DLPFC FC of nine DLPFC coordinates with the estimated clinical efficacy scores and then generated an overlapped map (Figure 7). These results showed that the association between FC and clinical efficacy was largely contributed by the midline sgACC, including: (a) the midline sgACC showed stronger negative FC with the Herbsman's responders' target than that of the nonresponders' target (Figure 7a); (b) the midline sgACC showed stronger negative FC with Fitzgerald's more effective target than that of the less effective target (Figure 7b); and (c) the midline sgACC‐DLPFC FC showed significant negative correlation with estimated clinical efficacy scores across the nine DLPFC sites (Figure 7c). As shown in Figure 7d, the overlapped voxels of the three statistical maps after thresholded at uncorrected *p* < .05 did not contain the right sgACC (the original sgACC coordinate: *x* = 6, *y* = 16, and *z* = −10 in Fox's study).

## 4 DISCUSSION

We systematically investigated the resting‐state functional connectivity between the ACCs (both pgACC and sgACC) with the left DLFPC, that is, the stimulation target area for the rTMS treatment of MDD. While the analyses and general results were similar as those in the study by Fox et al. (2012), two new findings were found in the current study as discussed below.

### 4.1 The pgACC may also be an effective region of rTMS

Albeit the results of many imaging studies support the sgACC as a critical region of MDD (Downey et al., 2016; Ho et al., 2014; Jaworska et al., 2014; Liu et al., 2015), including the FC findings of baseline sgACC‐DLPFC and sgACC‐left superior medial frontal gyrus connectivity predicted subsequent clinical improvement (Baeken et al., 2014; Cash et al., 2019; Fox et al., 2012; Ge, Downar, Blumberger, Daskalakis, & Vila‐Rodriguez, 2020; Liston et al., 2014; Weigand et al., 2018), there have a lot of evidences supporting the pgACC as a critical region of MDD (Ball, Stein, & Paulus, 2014; Boes et al., 2018; Pizzagalli, 2010; Silverstein et al., 2015; Zhou et al., 2017), and pgACC has been reported to show higher FC with the left lateral parietal cortex (IPL) at baseline in better clinical response group (Ge et al., 2020). In view of the above FC‐based researches, the current study investigated the ACC‐DLPFC FC as well as its association with rTMS efficacy. Similar as the sgACC, the pgACC also exhibited anticorrelation with the left DLFPC (Figure 2). Furthermore, for Herbsman's pair of targets, the negative FC of ACC‐DLPFC was stronger for responders' target than the nonresponders' (Figure 3). These results indicate that, similar as the sgACC, the pgACC may be a potential effective region of rTMS, that is, the rTMS stimulation on the left DLPFC probably takes effect on the pgACC via DLPFC‐pgACC FC.

### 4.2 The midline sgACC, instead of the right sgACC: potential effective region of rTMS on the left DLFPC for MDD treatment

For the sgACC, although we generally replicated the results of Fox et al. (2012), there is an apparent paradox for the sgACC‐DLPFC FC laterality: the sgACC was in the right side (*x* = 6, *y* = 16, and *z* = −10, the crosshair in Figure 7d) and the rTMS target was on the left DLPFC. As shown in the Section 3.5, we added a voxel‐wise analysis of association of sgACC‐DLPFC in the medial sgACC ROI centered at the coordinates (*x* = 0, *y* = 16, and *z* = −10, 20 mm radius) instead of (*x* = 6, *y* = 16, and *z* = −10). Results demonstrated that significant associations between FC and clinical efficacy were clustered around the midline sgACC (Figure 7). However, the voxel in the right sgACC (the original sgACC coordinate: *x* = 6, *y* = 16, and *z* = −10 in Fox's study) fell outside of the overlapping area. It means that, although the mean time course of the 10‐mm ROI centered at the right sgACC showed significant results (two *t* tests and one correlation analysis), the voxel per se did not. Instead, the voxels along the midline sgACC showed significant association between FC and clinical efficacy.

We did not repeat Section 2.2.6 in the voxel‐wise way. The first reason was that neither the 10‐mm radius ROI nor the 5‐mm radius ROI showed significant correlation between sgACC‐DLPFC FC with the rTMS efficacy in our dataset. Although Fox et al. found a significant correlation between sgACC‐DLPFC FC and clinical efficacy of the 27 patients (*r* = −.355, *p* < .05, one‐tailed correlation analysis in Fox's paper), there was an outlier which approximately two times of the standard deviation of the *r* values as shown in the original Figure S3b of Fox's paper (Fox et al., 2012). So, we extracted all the values of the *x*‐axis (i.e., *r* values) from Fox's Figure S3b and performed Pearson correlation analysis without this extreme *r* value. All the values of *y*‐axis (i.e., MADRS improvement) were from Paillere Martinot's study (Paillere Martinot et al., 2010). The correlation results became nonsignificant (*r* = −.210, *p* = .304) (Figure S1). It means that the predictive function on clinical efficacy of the ACC‐DLPFC FC in Fox's study might be largely driven by an outlier. Another reason was that unlike the nine DLPFC sites of which each value of coordinates was from averaged results of each study group, the 27 targets were from 27 individual patients. Hence, the 27 individuals may show larger variability.

### 4.3 The implications for rTMS treatment on MDD

Although some studies support only sgACC or only pgACC as critical node of MDD, a few studies indicated that both the sgACC and the pgACC could be critical nodes for MDD (Drevets, 2000; Pizzagalli, 2010). The current results indicate that both the midline sgACC and the pgACC could be potential effective regions for rTMS on MDD. The future rTMS treatment study on MDD may consider the following steps: (a) RS‐fMRI scanning before rTMS treatment; (b) the midline sgACC (or the left sgACC) could be taken as seed ROI and then their FC with the left DLPFC should be performed; (c) define the peak FC in the DLPFC for each patient; and (d) take the peak FC voxel in the DLPFC as individual stimulation target.

## 5 CONCLUSION

Either the midline sgACC (rather than the right sgACC) or the pgACC could be taken as effective region of rTMS on the left DLPFC for MDD treatment. The ACC‐DLPFC resting‐state functional connectivity can be considered to guide individualized precise localization of rTMS stimulation target on the left DLPFC in depression treatment.

## 6 LIMITATIONS

One limitation is that the current study only analyzed the RS‐fMRI data of the healthy subjects. It would be more reliable and more helpful on data from MDD patients, ideally, on the RS‐fMRI data before and after rTMS treatment. Another limitation is that there has been lack of strong evidence for either the pgACC or the sgACC as a critical node. Future functional neuroimaging studies should focus on this topic and reveal individual abnormality in either the pgACC or sgACC.

## CONFLICT OF INTERESTS

All authors declare that they have no conflict of interest.

## AUTHOR CONTRIBUTION

Y.F.Z. and J.W involved in experimental design. Y.J., N.Z., X.P.D., and Z.J.F involved in data collection. Y.J., G.F.H., and M.M. involved in data analyses. Y.J., Y.F.Z., and J.W wrote the article.

## Supporting information

Figure S1

Figure S2

Figure S3

Figure S4

Supplementary Material

Supplementary Material

## Data Availability

The data that support the findings of this study are available from the corresponding author upon reasonable request.
