# Supplementary figures and images for: Pregenual or subgenual anterior cingulate cortex as potential effective region for brain stimulation of depression
Source: Brain Behav. 2020 Mar 8;10(4):e01591. doi: 10.1002/brb3.1591 (PMC7177590; doi:10.1002/brb3.1591)

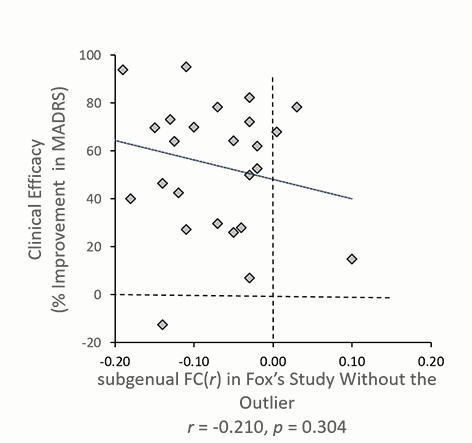

Supplement: Supplementary file 1 — Figure S1 [file BRB3-10-e01591-s001.tif]

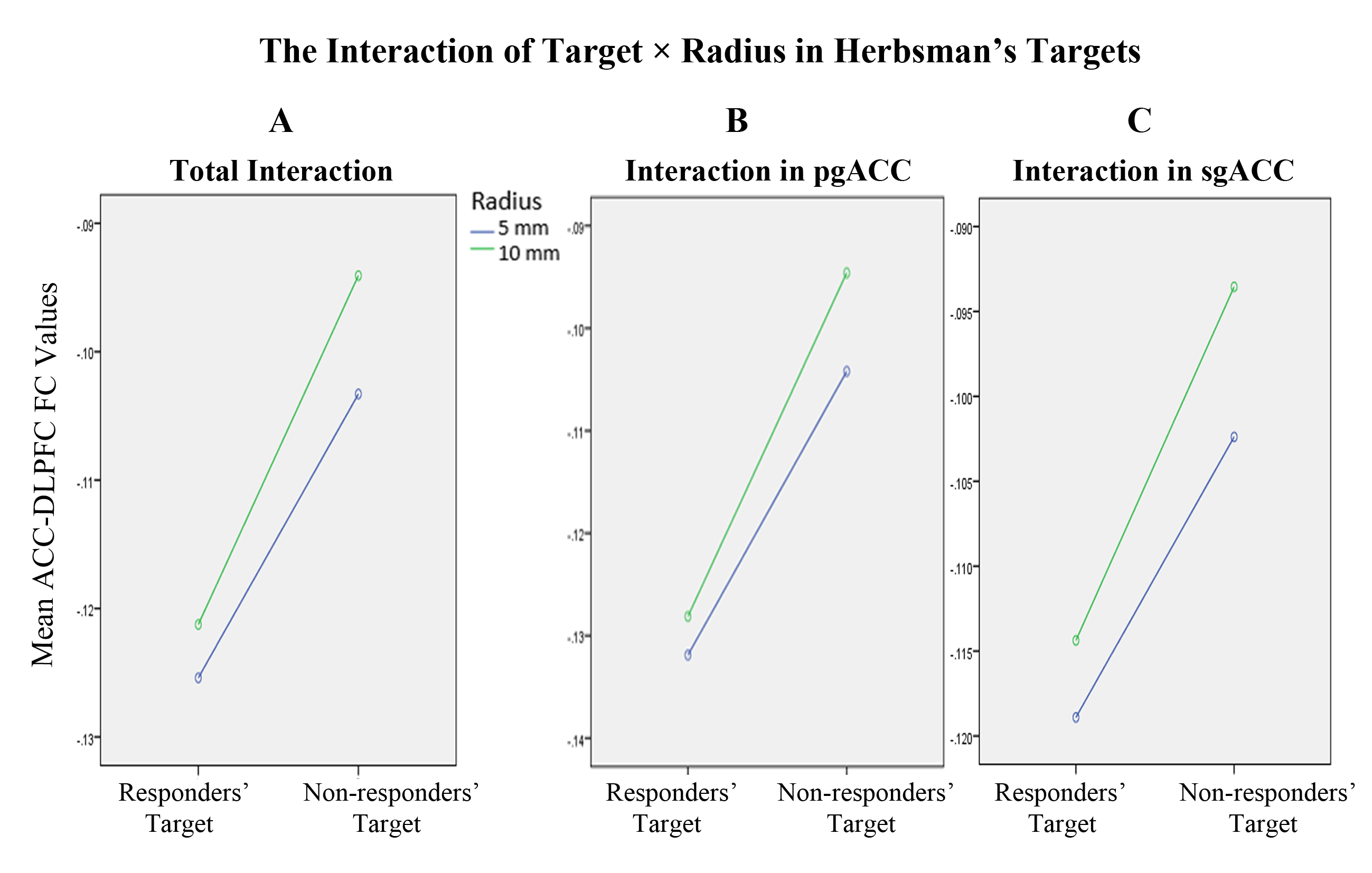

Supplement: Supplementary file 2 — Figure S2 [file BRB3-10-e01591-s002.tif]

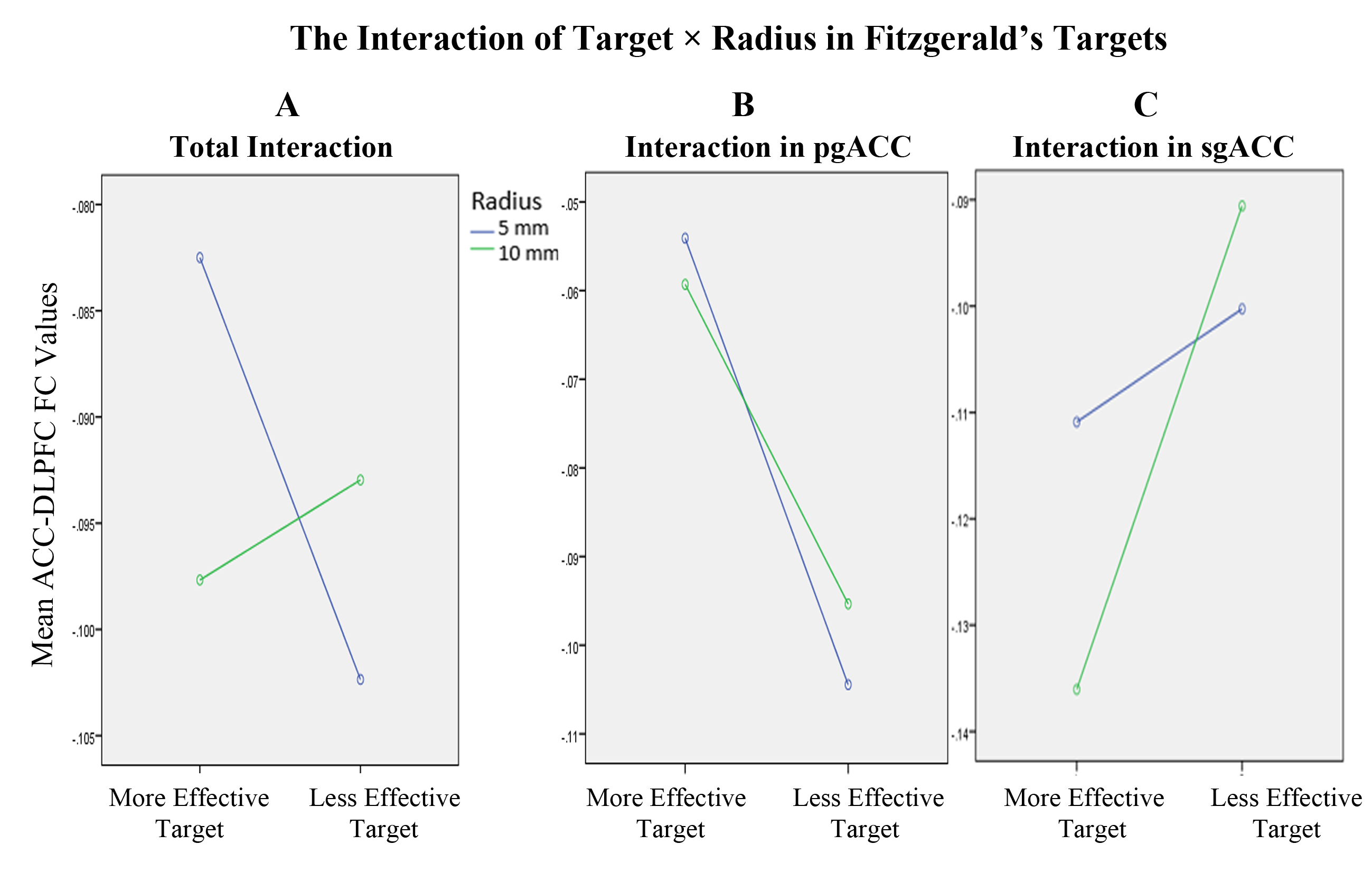

Supplement: Supplementary file 3 — Figure S3 [file BRB3-10-e01591-s003.tif]

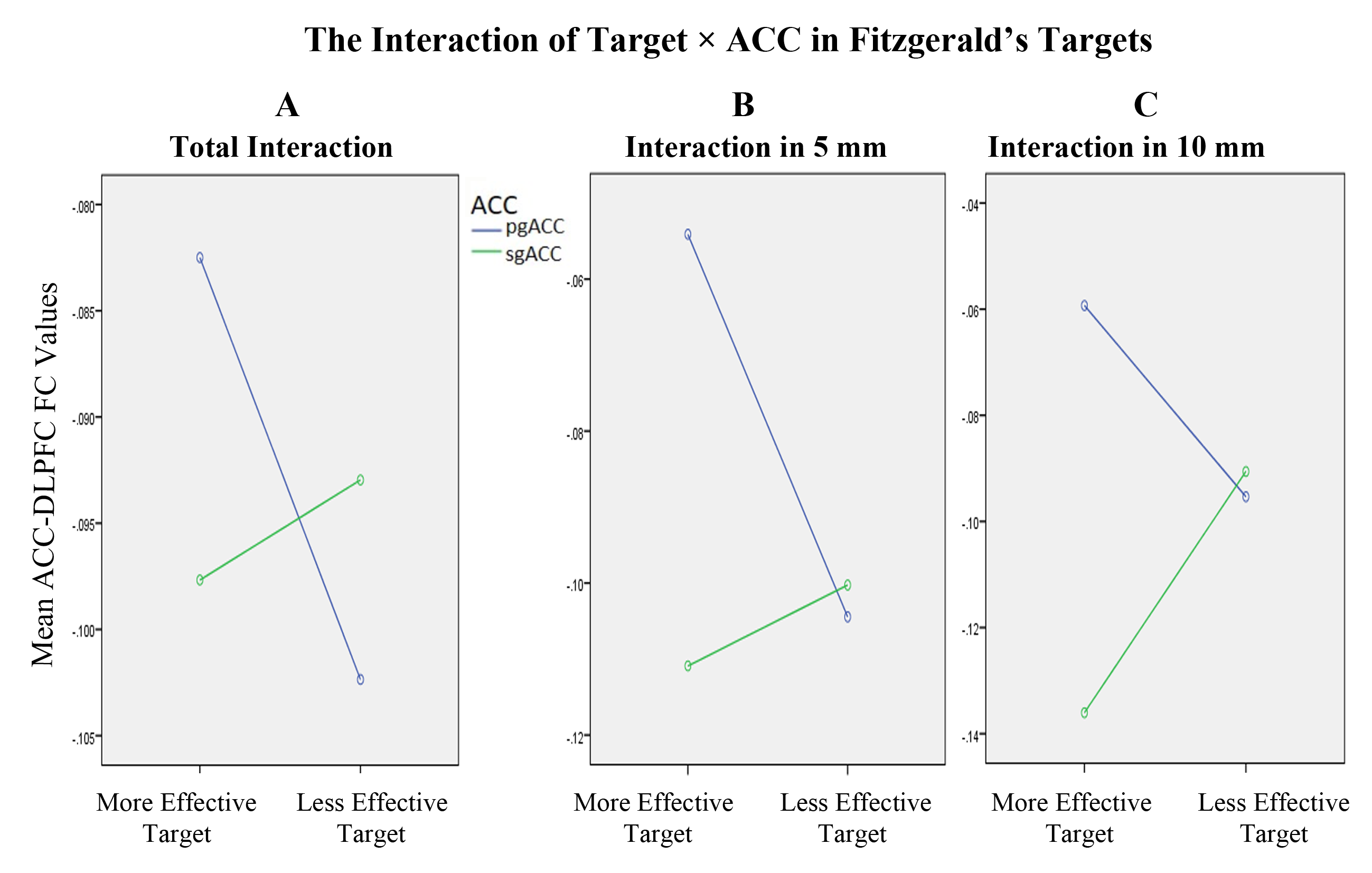

Supplement: Supplementary file 4 — Figure S4 [file BRB3-10-e01591-s004.tif]
