# Supplementary material for: Pregenual or subgenual anterior cingulate cortex as potential effective region for brain stimulation of depression
Source: Brain Behav. 2020 Mar 8;10(4):e01591. doi: 10.1002/brb3.1591 (PMC7177590; doi:10.1002/brb3.1591)
Supplement: Supplementary file 5 — Supplementary Material [file BRB3-10-e01591-s005.docx]

**Pregenual or Subgenual Anterior** **Cingulate Cortex as Potential Effective Region for Brain Stimulation of Depression**

**Supporting Information**

**Supporting Methods**

**Analysis of Variance (ANOVA) on anterior cingulate cortex (ACC)-dorsal lateral prefrontal cortex (DLPFC) functional connectivity (FC) difference**

To reveal the ACC-DLPFC FC difference, the mean FC values of the 88 healthy participants were entered into a repeated measures 2 × 2 × 2 (target type: better efficacy vs. worse efficacy; ACC type: pregenual ACC vs. subgenual ACC; radius type: 5 mm vs. 10 mm) ANOVA. In our study, there were two pairs of DLPFC targets which were previously shown to differ in clinical efficacy, so the 3-way repeated-measure ANOVA was performed in the pair of Herbsman’s targets (responders vs. non-responders) and the pair of Fitzgerald’s targets (more effective vs. less effective) separately.

The main effects of radius and ACC were not significant (Table S1). In the Herbsman’s pair of targets (responders and non-responders), the ANOVA results revealed a significant main effect on target factor (*F*_1, 87_ = 22.496, *p* = 8.0 × 10^-6^) (Table S1). After pairwise comparisons, we found that the FC strength of ACC-DLPFC was stronger in the responders’ target than in the non-responders’ target in all subgroups (see Table S2 and Figure S2). In terms of interaction effect, there was a significant 2-way interaction of target × radius (*F*_1, 87_ = 7.449, *p* = 0.008, Table S1). To figure out the meaning of this interaction, we did 2-way ANOVA (target type: responders vs. non-responders; radius type: 5 mm vs. 10 mm) for the subgenual ACC (sgACC) and pregenual ACC (pgACC) separately. The results showed that the interaction of target × radius only presented under the level of pgACC (Table S3). After the simple effect analyses, we found that this interaction was mainly contributed by the difference between 5-mm radius pgACC-DLPFC FC and 10-mm radius pgACC-DLPFC FC in the non-responders’ target (Table S4, and Figure S2B, C), but this difference was not significant. Further, because this stronger contribution was from non-responder’s target, it is therefore less of practical importance.

When referring to the Fitzgerald’s pair of targets, the 3-way repeated-measures ANOVA revealed no significant main effects of target, ACC, or radius (Table S5). The pairwise comparisons showed that only the 10-mm radius sgACC had stronger negative FC with more effective target than less effective target (*F*_1, 87_ = 5.032, *p* = 0.027) (Table S6). This result was similar as Fox’s finding. Significant 2-way interaction was found in target × radius (*F*_1, 87_ = 10.631, *p* = 0.002) and target × ACC (*F*_1, 87_ = 5.005, *p* = 0.028) (Table S5, Figure S3 and Figure S4). Then we did 2-way ANOVA in these 2 interaction effects to further explore the meaning of interaction in this study. In the interaction of target × radius, the significant interaction effect was only shown in the sgACC level but not shown in the pgACC level (Table S7). The simple effect analyses showed stronger FC of 10-mm radius sgACC-DLPFC than 5-mm radius in the more effective target, but no significant difference was found in the less effective target (Table S8, and Figure S3B, C). In terms of target × ACC interaction, the significant interaction effect only existed in the 10-mm radius level (Table S7). The results of simple effect analyses showed that, in the more effective target, the FC of 10-mm radius sgACC-DLPFC was negatively stronger than pgACC, but there was no significant difference in the less effective target (Table S8, and Figure S4B, C).

**Table S1.** The 3-way repeated-measures ANOVA results of ACC-DLPFC FC values among Target (responders vs. non-responders’ target in Herbsman’s study), Radius (5 mm vs. 10 mm) and ACC (pgACC vs. sgACC)

| Sources | *F* Values | *p* Values |
| --- | --- | --- |
| Target | 22.496 | 8.0 × 10^-6^** |
| Radius | 1.511 | 0.222 |
| ACC | 0.130 | 0.719 |
| Target × Radius | 7.449 | 0.008** |
| Target × ACC | 2.957 | 0.089 |
| Radius × ACC | 1.5 × 10^-4^ | 1.000 |
| Target × Radius × ACC | 0.198 | 0.657 |

ANOVA, analysis of variance; ACC, anterior cingulate cortex; DLPFC, dorsal lateral prefrontal cortex; FC, functional connectivity; pgACC, pregenual ACC; sgACC, subgenual ACC; **p* < 0.05, ***p* < 0.01.**Table S2.** The follow-up pairwise comparisons of ACC-DLPFC FC between responders and non-responders’ target in Herbsman’s study

| Radius | ACC | Target | Mean FC | *F* Values | *p* Values |
| --- | --- | --- | --- | --- | --- |
| 5 mm | pgACC | Responder | -0.132 | 13.227 | 4.7 × 10^-4^** |
|  |  | Non-responder | -0.104 |  |  |
|  | sgACC | Responder | -0.119 | 11.372 | 1.1 × 10^-3^** |
|  |  | Non-responder | -0.102 |  |  |
| 10 mm | pgACC | Responder | -0.128 | 19.883 | 2.4 × 10^-5^** |
|  |  | Non-responder | -0.095 |  |  |
|  | sgACC | Responder | -0.114 | 17.984 | 5.5 × 10^-5^** |
|  |  | Non-responder | -0.094 |  |  |

ACC, anterior cingulate cortex; DLPFC, dorsal lateral prefrontal cortex; FC, functional connectivity; pgACC, pregenual ACC; sgACC, subgenual ACC; **p* < 0.05, ***p* < 0.01.

**Table S3.** The 2-way ANOVA results of ACC-DLPFC FC values among Target (responders vs. non-responders’ target in Herbsman’s study) and Radius (5 mm vs. 10 mm) in 2 subgroup of ACC levels.

| Sources | | *F* Values | *p* Values |
| --- | --- | --- | --- |
| pgACC | Target | 16.811 | 9.3 × 10^-5^** |
|  | Radius | 1.027 | 0.314 |
|  | Target × Radius | 5.603 | 0.020* |
| sgACC | Target | 15.597 | 1.6 × 10^-4^** |
|  | Radius | 0.781 | 0.379 |
|  | Target × Radius | 2.710 | 0.103 |

ANOVA, analysis of variance; ACC, anterior cingulate cortex; DLPFC, dorsal lateral prefrontal cortex; FC, functional connectivity; pgACC, pregenual ACC; sgACC, subgenual ACC; **p* < 0.05, ***p* < 0.01.

**Table S4.** The results of simple effect analyses on the interaction of target × radius in Herbsman’s targets

|  | pair-wise comparisons | | *F* Values | | *p* Values |
| --- | --- | --- | --- | --- | --- |
| Total Interaction | Responders | 5 mm vs. 10 mm | 0.583 | 0.447 | |
|  | Non-responders | 5 mm vs. 10 mm | 2.713 | 0.103 | |
| Interaction in pgACC | Responders | 5 mm vs. 10 mm | 0.353 | 0.554 | |
|  | Non-responders | 5 mm vs. 10 mm | 1.851 | 0.177 | |
| Interaction in sgACC | Responders | 5 mm vs. 10 mm | 0.349 | 0.556 | |
|  | Non-responders | 5 mm vs. 10 mm | 1.325 | 0.253 | |

ACC, anterior cingulate cortex; pgACC, pregenual ACC; sgACC, subgenual ACC.

**Table S5.** The 3-way repeated-measures ANOVA results of ACC-DLPFC FC values among Target (more effective vs. less effective target in Fitzgerald’s study), Radius (5 mm vs. 10 mm) and ACC (pgACC vs. sgACC)

| Sources | *F* Values | *p* Values |
| --- | --- | --- |
| Target | 0.143 | 0.706 |
| Radius | 0.445 | 0.506 |
| ACC | 3.058 | 0.084 |
| Target × Radius | 10.631 | 0.002** |
| Target × ACC | 5.005 | 0.028* |
| Radius × ACC | 1.859 | 0.176 |
| Target × Radius × ACC | 2.003 | 0.161 |

ANOVA, analysis of variance; ACC, anterior cingulate cortex; DLPFC, dorsal lateral prefrontal cortex; FC, functional connectivity; pgACC, pregenual ACC; sgACC, subgenual ACC; **p* < 0.05, ***p* < 0.01.

**Table S6.** The follow-up pairwise comparisons of ACC-DLPFC FC between more effective and less effective target in Fitzgerald’s study

| Radius | ACC | Target | Mean FC | *F* Values | *p* Values |
| --- | --- | --- | --- | --- | --- |
| 5 mm | pgACC | More Effective | -0.054 | 2.620 | 0.109 |
|  |  | Less Effective | -0.104 |  |  |
|  | sgACC | More Effective | -0.111 | 0.255 | 0.615 |
|  |  | Less Effective | -0.100 |  |  |
| 10 mm | pgACC | More Effective | -0.059 | 1.430 | 0.235 |
|  |  | Less Effective | -0.095 |  |  |
|  | sgACC | More Effective | -0.136 | 5.032 | 0.027* |
|  |  | Less Effective | -0.091 |  |  |

ACC, anterior cingulate cortex; DLPFC, dorsal lateral prefrontal cortex; FC, functional connectivity; pgACC, pregenual ACC; sgACC, subgenual ACC; **p* < 0.05.

**Table S7.** The split 2-way ANOVA results of ACC-DLPFC FC values among Target × Radius and Target × ACC in Fitzgerald’s targets.

|  | Source | | *F* Value | *p* Value |
| --- | --- | --- | --- | --- |
| Target × Radius | pgACC | Target | 2.061 | 0.155 |
|  |  | Radius | 0.168 | 0.683 |
|  |  | Target × Radius | 1.565 | 0.214 |
|  | sgACC | Target | 1.941 | 0.167 |
|  |  | Radius | 1.489 | 0.226 |
|  |  | Target × Radius | 13.851 | 3.5 × 10^-4^** |
| Target × ACC | 5 mm | Target | 0.954 | 0.332 |
|  |  | ACC | 1.956 | 0.165 |
|  |  | Target × ACC | 3.183 | 0.078 |
|  | 10 mm | Target | 0.053 | 0.818 |
|  |  | ACC | 4.233 | 0.043* |
|  |  | Target × ACC | 6.884 | 0.010* |

ANOVA, analysis of variance; ACC, anterior cingulate cortex; pgACC, pregenual ACC; sgACC, subgenual ACC; DLPFC, dorsal lateral prefrontal cortex; FC, functional connectivity; **p* < 0.05, ***p* < 0.01.

**Table S8.** The results of simple effect analyses on the interaction of target × radius and target × ACC in Fitzgerald’s targets

| Interaction | | Pair-wise Comparisons | | *F* Vaules | *p* Values |
| --- | --- | --- | --- | --- | --- |
| Target × Radius | Total Interaction | More Effective | 5 mm vs. 10 mm | 6.567 | 0.012* |
|  |  | Less Effective | 5 mm vs. 10 mm | 2.856 | 0.095 |
|  | Interaction in pgACC | More Effective | 5 mm vs. 10 mm | 0.446 | 0.506 |
|  |  | Less Effective | 5 mm vs. 10 mm | 1.662 | 0.201 |
|  | Interaction in sgACC | More Effective | 5 mm vs. 10 mm | 9.736 | 0.002** |
|  |  | Less Effective | 5 mm vs. 10 mm | 1.584 | 0.212 |
| Target × ACC | Total Interaction | More Effective | pgACC vs. sgACC | 6.407 | 0.013* |
|  |  | Less Effective | pgACC vs. sgACC | 0.045 | 0.833 |
|  | Interaction in 5 mm | More Effective | pgACC vs. sgACC | 4.111 | 0.046* |
|  |  | Less Effective | pgACC vs. sgACC | 0.035 | 0.853 |
|  | Interaction in 10 mm | More Effective | pgACC vs. sgACC | 8.891 | 0.004** |
|  |  | Less Effective | pgACC vs. sgACC | 0.052 | 0.819 |

ACC, anterior cingulate cortex; pgACC, pregenual ACC; sgACC, subgenual ACC; **p* < 0.05, ***p* < 0.01.
