# Supplementary material for: Pregenual or subgenual anterior cingulate cortex as potential effective region for brain stimulation of depression
Source: Brain Behav. 2020 Mar 8;10(4):e01591. doi: 10.1002/brb3.1591 (PMC7177590; doi:10.1002/brb3.1591)
Supplement: Supplementary file 6 — Supplementary Material [file BRB3-10-e01591-s006.docx]

**Supporting information figure legends**

**Figure S1.** After delete the outlier which located beyond the 2 times SD of FC values in Fox’s study, the correlation between sgACC–DLPFC FC values and clinical efficacy scores became non-significant. ACC, anterior cingulate cortex; pgACC, pregenual ACC; sgACC, subgenual ACC; DLPFC, dorsal lateral prefrontal cortex; FC, functional connectivity; MADRS, Montgomery Asberg Depression Rating Scale.

**Figure S2.** The interaction effect of target × radius in Herbsman’s targets. ACC, anterior cingulate cortex; DLPFC, dorsal lateral prefrontal cortex; FC, functional connectivity.

**Figure S3.** The interaction effect of target × radius in Fitzgerald’s targets. ACC, anterior cingulate cortex; DLPFC, dorsal lateral prefrontal cortex; FC, functional connectivity.

**Figure S4.** The interaction effect of target × ACC in Fitzgerald’s targets. ACC, anterior cingulate cortex; pgACC, pregenual ACC; sgACC, subgenual ACC; DLPFC, dorsal lateral prefrontal cortex; FC, functional connectivity.
